# Anaerococcus vaginalis-Induced Acute Scrotal Abscess: A Case Report

**DOI:** 10.7759/cureus.114808

**Published:** 2026-08-19

**Authors:** Prudhvi Gundupalli, Farhood Salehi, Chandni Davi, Fadi Adnan, Maryam Adnan, Zain Saadaldin, Mohammed Bahaa AlDeen

**Affiliations:** 1 School of Medicine, Texas Tech University Health Sciences Center, Lubbock, USA; 2 Department of Internal Medicine, Texas Tech University Health Sciences Center, Amarillo, USA; 3 College of Natural Sciences, The University of Texas at Austin, Austin, USA; 4 School of Natural Sciences and Mathematics, The University of Texas at Dallas, Richardson, USA; 5 Department of Biological Sciences, Texas Tech University, Lubbock, USA; 6 Department of Internal Medicine, Northwest Texas Healthcare System, Amarillo, USA

**Keywords:** anaerobic infection, anaerococcus vaginalis, culture-guided therapy, fournier's gangrene, scrotal abscess, urogenital infection

## Abstract

Fournier’s gangrene is a rapidly progressive necrotizing soft tissue infection of the perineum that is typically polymicrobial and is associated with substantial morbidity and mortality. Anaerococcus vaginalis is a rare anaerobic organism that has rarely been identified as a causative agent in necrotizing infections. To our knowledge, this organism has not previously been reported as the primary pathogen causing a scrotal abscess progressing to Fournier’s gangrene. A 63-year-old male with a past medical history of alcoholic dementia presented to an urgent care facility with left hemiscrotal pain and swelling, which was identified as a hemiscrotal abscess. He underwent incision and drainage on site. Four days later, positive wound cultures prompted referral to the emergency department for further evaluation and management. Persistent pain and swelling prompted repeat imaging that revealed a 2.6-cm gas-containing subdermal collection within the left hemiscrotum concerning for necrotizing infection. Emergent operative exploration confirmed Fournier’s gangrene, and debridement and excision of the scrotal wall cysts were performed. Intraoperative cultures grew A. vaginalis. The patient denied any sexual activity in the preceding 12 months. Empiric broad-spectrum antibiotics were initiated postoperatively and subsequently de-escalated to culture-directed oral therapy, which resulted in clinical improvement and discharge from the hospital. This case represents, to our knowledge, the first reported case of A. vaginalis as the primary pathogen in a scrotal abscess progressing to Fournier’s gangrene. This report highlights the importance of early recognition, maintaining a broad differential for necrotizing infections, prompt surgical intervention, and culture-guided antibiotic management to optimize patient outcomes.

## Introduction

Fournier’s gangrene is a rapidly progressive necrotizing soft tissue infection of the perineum and scrotum that is most often classified as polymicrobial, typically involving a combination of Gram-positive cocci, fermenting and nonfermenting bacilli, and anaerobic organisms [1]. Other microbiological etiologies include monomicrobial infections with *Streptococcus *spp. or *Staphylococcus aureus*, *Vibrio *spp. or *Aeromonas *spp. infections, and fungal infections [1]. It predominantly affects immunocompromised or debilitated individuals, including those with diabetes mellitus, alcoholism, malignancy, or other forms of immune suppression. Delayed presentation and impaired host immunity are known to contribute to the severity and mortality of the disease, which can exceed 30% in some cases despite management [2,3].

Among the anaerobic pathogens implicated in necrotizing infections, *Anaerococcus vaginalis* is rarely identified as a causative agent. To date, no published cases have reported *A. vaginalis* as the causative organism in Fournier’s gangrene or an isolated scrotal abscess. However, parallels exist with other anaerobic urogenital pathogens, such as *Gardnerella vaginalis *and *Prevotella bivia*, which have been implicated in male perineal abscesses and severe genital infections, particularly in settings of impaired hygiene, immune dysfunction, or anatomical disruption [4-6].

*A. vaginalis *is a Gram-positive, non-spore-forming, obligate anaerobic coccus that colonizes the gastrointestinal tract, skin, and genitourinary mucosa. It is a member of the *Anaerococcus *genus, which includes opportunistic pathogens capable of causing deep-seated infections, often in polymicrobial contexts [7,8]. While *Anaerococcus *species have been isolated from pelvic abscesses, diabetic foot infections, and decubitus ulcers, specific reports of *A. vaginalis *as a primary pathogen remain scarce [9].

Scrotal and perineal infections due to anaerobes are frequently underrecognized because anaerobic cultures require specific transport conditions and are often omitted in the initial workup. The introduction of matrix-assisted laser desorption/ionization time-of-flight mass spectrometry (MALDI-TOF MS) has enhanced the ability to rapidly and accurately identify uncommon anaerobic organisms such as *A. vaginalis*, which previously might have gone undetected or been misclassified [10].

This report aims to present the first documented case of Fournier’s gangrene caused by *A. vaginalis*, contribute to the expanding spectrum of clinically significant infections involving *A. vaginalis*, and underscore the importance of considering anaerobic organisms in the differential diagnosis of necrotizing scrotal infections, especially in patients with altered immune status or social vulnerability. Early surgical intervention, broad-spectrum antibiotic coverage, including anti-anaerobic agents, and microbiologic confirmation remain key in management.

## Case presentation

**Figure 1:**
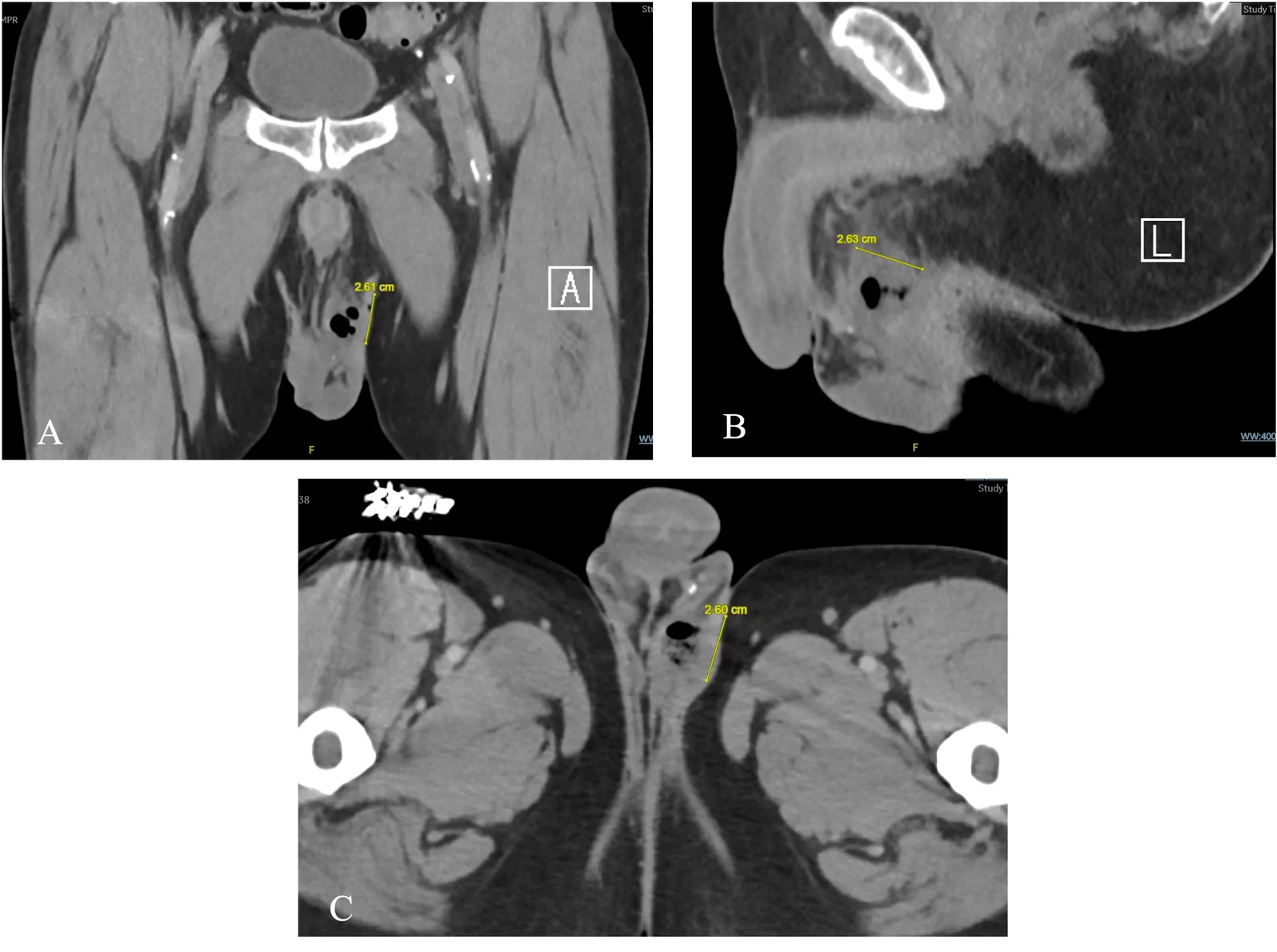
CT of the abdomen/pelvis with contrast showing a 2.6-cm gas-containing left hemiscrotal subdermal collection indicative of infection in coronal (A), sagittal (B), and transverse (C) views.

A 63-year-old male with a medical history of alcoholic dementia, alcoholic neuropathy, and restless leg syndrome presented to the urgent care facility with left scrotal pain and swelling. Examination and imaging confirmed a hemiscrotal abscess. An incision and drainage of the scrotum was performed, and the patient was subsequently discharged. A wound culture obtained at that time grew *Staphylococcus hominis*. Given the patient’s medical history of alcoholic dementia, the patient’s family was notified of the results, and the patient was brought to the emergency department for IV antibiotic therapy. A CT of the abdomen and pelvis with contrast in the hospital ED revealed a 2.6-cm gas-containing left hemiscrotal subdermal collection concerning for a gas-forming infection (Figure 1), as well as a 1-cm right posterior hemiscrotal collection or a sebaceous cyst. These findings were concerning for Fournier’s gangrene, and the patient was taken to the operating room the same day for debridement and excision of two scrotal wall cysts. Intraoperative cultures were obtained, and a Penrose drain was placed. The diagnosis of Fournier’s gangrene was confirmed intraoperatively.

On hospital day (HOD) 2, the patient was admitted to the medical service and started empirically on IV vancomycin and piperacillin-tazobactam to ensure broad-spectrum coverage of Gram-positive, Gram-negative, and anaerobic organisms. Gram stain of the intraoperative scrotal collection showed moderate gram-positive cocci in pairs. By HOD 3, intraoperative anaerobic cultures had grown heavy *A. vaginalis*, identified via MALDI-TOF MS. Susceptibility testing for this organism was not performed. On HOD 4, intraoperative cultures further revealed light growth of *Actinomyces neuii*.

Broad-spectrum antibiotic coverage with vancomycin and piperacillin-tazobactam was de-escalated to oral clindamycin and metronidazole over HOD 5-6 based on the final culture report. By HOD 7, the patient demonstrated significant clinical improvement, with improved pain control. On HOD 8, he was discharged home in improved condition with family support on oral clindamycin and metronidazole. The patient was scheduled for follow-up but was subsequently lost to follow-up after hospital discharge.

## Discussion

This case illustrates that *A. vaginalis* can serve as a primary pathogen in the development of Fournier’s gangrene, also highlighting the importance of empiric anaerobic coverage in cases of unusual cellulitis and abscess. To our knowledge, this case documents the first instance of *A. vaginalis *as the primary pathogen causing a scrotal abscess, further underscoring the need to remain vigilant for uncommon anaerobic organisms in the development of such infections. Notably, our case is unique in that heavy growth of* A. vaginalis *on culture, paired with little to no growth of other microbes, led us to identify *A. vaginalis *as the causative organism in a patient who had not been sexually active for the preceding 12 months, suggesting that his infection may have derived from local colonization or other potential host factors.

*A. vaginalis *is increasingly recognized among bacteria isolated from a range of polymicrobial infectious conditions, most notably including bacterial vaginosis (BV), chronic wounds, and other soft tissue infections [11,12]. In the case of BV, *A. vaginalis* plays a contributory role in the pathogenesis of the disease by participating in the shift from a *Lactobacillus*-dominated flora to a diverse, anaerobe-rich community. This imbalance allows organisms such as *A. vaginalis *to proliferate and integrate into biofilms along with bacteria such as *G. vaginalis*, *P. bivia*, and *Atopobium vaginae *[13]. Biofilm formation is a key virulence mechanism, as it enables bacteria to adhere to vaginal epithelial cells, evade host defenses, and resist antimicrobial therapy. These pathogenic mechanisms illustrate how *A. vaginalis*, though often overlooked, may serve as an important cofactor in both gynecologic and systemic anaerobic infections. In this instance, the lack of prior sexual activity highlights the organism’s capacity to cause uncommon genital infections and reinforces the need to maintain a broad differential when encountering atypical cellulitis or abscess presentations.

Although susceptibility testing was not performed for this particular organism in our report, prior studies have demonstrated that *Anaerococcus *species are 100% susceptible to amoxicillin, piperacillin, imipenem, vancomycin, linezolid, and metronidazole [14]. The species are additionally 80% susceptible to cefotaxime, clindamycin, moxifloxacin, and rifampicin [14]. Our team used an empiric combination of vancomycin and piperacillin-tazobactam, with both agents effectively covering this organism. While resistance to clindamycin has been reported, our team’s decision to use combination therapy with the addition of metronidazole mitigated this risk and ensured appropriate home treatment [14].

The differential diagnosis of acute scrotal pathology with subcutaneous gas deserves careful consideration, as not all cases of scrotal emphysema are infectious in origin [15]. Traumatic, iatrogenic, or substance-related etiologies can produce radiological findings indistinguishable from early Fournier’s gangrene, particularly in patients with impaired cognition or an unreliable history [15]. In the present case, the patient’s underlying alcoholic dementia and denial of recent sexual activity underscored the importance of maintaining a broad differential while pursuing prompt surgical exploration. Clinicians should remain vigilant that the absence of classic risk factors does not exclude necrotizing fasciitis and, conversely, that gas-containing scrotal collections may occasionally arise from noninfectious mechanisms requiring a distinct management approach.

Fournier’s gangrene is frequently due to polymicrobial infection, and anaerobic coverage is essential to reduce the risk of adverse outcomes [2,3]. Broad-spectrum antibiotics with anaerobic coverage remain the cornerstone of initial management of these infections. Physicians should prioritize utilizing intraoperative cultures to improve clinical outcomes and uphold antibiotic stewardship, as they can narrow agent selection while ensuring effective coverage.

## Conclusions

This case highlights a rare presentation of Fournier’s gangrene caused by* A. vaginalis*, expanding the current understanding of its pathogenic potential beyond the urogenital tract and the context of recent sexual activity. Early surgical intervention, prompt initiation of broad-spectrum antibiotics with anaerobic coverage, and microbiologic confirmation were instrumental in achieving a favorable treatment outcome. As the first reported case of *A. vaginalis *as the primary pathogen in a scrotal abscess, this report underscores the importance of considering atypical anaerobic organisms in necrotizing perineal infections and reinforces the clinical value of thorough culture-driven management.
